# Analysis of inflammation-related nigral degeneration and locomotor function in *DJ-1*^*−/−*^ mice

**DOI:** 10.1186/1742-2094-10-50

**Published:** 2013-04-28

**Authors:** Thi A Nguyen, Tamy Frank-Cannon, Terina N Martinez, Kelly A Ruhn, Marian Marvin, Bradford Casey, Isaac Treviño, John J Hong, Matthew S Goldberg, Malú G Tansey

**Affiliations:** 1 Department of Physiology, The University of Texas Southwestern Medical Center, 5323 Harry Hines Blvd, Dallas, TX 75390, USA; 2 Department of Neurology and Neurotherapeutics, The University of Texas Southwestern Medical Center, 5323 Harry Hines Blvd, Dallas, TX 75390, USA; 3 Department of Psychiatry, The University of Texas Southwestern Medical Center, 5323 Harry Hines Blvd, Dallas, TX 75390, USA; 4 Department of Physiology, Emory University School of Medicine, 615 Michael Street, Atlanta, GA, 30322, USA

**Keywords:** DJ-1, Dopamine, LPS, Macrophage, Neuroinflammation, Neurodegeneration, Oxidative stress, TNF

## Abstract

**Background:**

Complex interactions involving genetic susceptibility and environmental factors are thought to underlie the pathogenesis of Parkinson’s disease (PD). Although the role of inflammatory processes in modulating risk for development of PD has yet to be fully understood, prospective studies suggest that chronic use of NSAIDs reduce the incidence of PD. Loss-of-function mutations in the *DJ-1* gene cause a rare form of familial PD with an autosomal recessive pattern of inheritance; however, *DJ-1−/−* mice do not display nigrostriatal pathway degeneration, suggesting that additional factors such as inflammation may be needed to induce neurodegeneration on the background of *DJ-1* gene mutations. Neuroinflammation causes oxidative stress and, based on evidence that DJ-1 plays a protective role against oxidative stress, we investigated whether *DJ-1−/−* mice display increased vulnerability to inflammation-induced nigral degeneration.

**Methods:**

We exposed adult wild-type and *DJ-1−/−* mice to repeated intranasal administration of soluble TNF (inTNF) or repeated intraperitoneal injections of low-dose lipopolysaccharide (LPS) or saline vehicle. We measured locomotor performance using a variety of behavior tasks, striatal dopamine (DA) content by HPLC, DA neuron (TH+ cells) and total neuron (NeuN+ cells) number in the substantia nigra pars compacta and ventral tegmental area by unbiased stereology, number of Iba1-positive microglia, and mRNA levels of inflammatory and oxidative stress genes by quantitative PCR in the midbrain, cortex and isolated peritoneal macrophages of *DJ-1−/−* and wild-type mice.

**Results:**

We found that chronic LPS injections induced similar neuroinflammatory responses in the midbrains of *DJ-1−/−* mice and wild-type mice and neither group developed locomotor deficits or nigral degeneration. inTNF administration did not appear to induce neuroinflammatory responses in LPS-treated wild-type or *DJ-1−/−* mice. The lack of vulnerability to inflammation-induced nigral degeneration was not due to enhanced anti-oxidant gene responses in the midbrains of *DJ-1−/−* mice which, in fact, displayed a blunted response relative to that of wild-type mice. Peripheral macrophages from wild-type and *DJ-1−/−* mice displayed similar basal and LPS-induced inflammatory and oxidative stress markers *in vitro.*

**Conclusions:**

Our studies indicate that *DJ-1−/−* mice do not display increased vulnerability to inflammation-related nigral degeneration in contrast to what has been reported for 1-methyl-4-phenyl-1,2,3,6-tetrahydropyrindine. We conclude that either DJ-1 does not have a critical role in protecting DA neurons against inflammation-induced oxidative stress and/or there is compensatory gene expression in the midbrain of *DJ-1−/−* mice that renders them resistant to the cytotoxic effects triggered by chronic peripheral inflammation.

## Background

Parkinson’s disease (PD) is the most common neurodegenerative movement disorder. It is an age-dependent disease characterized by a severe depletion of dopamine (DA)-producing neurons in the substantia nigra pars compacta (SNpc) that project to the striatum. The gradual loss of nigrostriatal pathway function results in slowly progressing clinical symptoms including tremor, rigidity and slowness of movement. Despite intensive research, the causes of PD remain unknown. Over the last decade, a vast number of published studies have indicated that inflammation-derived oxidative stress and cytokine-dependent neurotoxicity are likely to contribute to nigrostriatal pathway degeneration [1-5], the pathological hallmark of PD. Specifically, post-mortem analyses of brains from PD patients confirmed the presence of inflammatory mediators in the area of the SNpc where maximal destruction of melanin-containing DA-producing neurons occurs [6-11]. Signs of inflammation included activated microglia and accumulation of cytokines (including TNF, IL-1β, IL-6, and IFNγ), some of which exert neurotoxic effects on DA neurons [5]. In addition to extrinsic factors, SNpc dopaminergic neurons may be uniquely vulnerable to neuroinflammatory insults that enhance cellular oxidative stress. For example, the higher sensitivity of nigral DA neurons to injury induced by neuroinflammatory mediators may be secondary to a reduction of endogenous anti-oxidant capacity (such as glutathione depletion). Pharmacologically, chronic infusion of various anti-inflammatory compounds (including COX-2-selective NSAIDs or soluble TNF-selective inhibitors) rescues nigral DA neurons from progressive degeneration and death [12-14]. These findings raise the interesting possibility that environmental triggers may initiate cytokine-driven neuroinflammation and may contribute to the development of PD in humans.

Monogenic forms of PD have been linked to loss-of-function mutations in a number of genes, giving rise to autosomal recessive parkinsonism [15], including mutations in *parkin*, which encodes an E3 ligase, and in *DJ-1*, which encodes a putative redox sensor that associates with chaperones [16] and translocates to mitochondria during conditions of oxidative stress [17-22]. In addition to its proposed role as a redox sensor [23], DJ-1 may also have important functions as an RNA binding protein [24]. Although *DJ-1−/−* mice have been reported to be hypersensitive to the neurotoxin 1-methyl-4-phenyl-1,2,3,6-tetrahydropyrindine (MPTP) [25], and to display abnormalities in dopaminergic function when exposed to the herbicide paraquat [26], these mice do not develop nigrostriatal degeneration in the absence of stresses [17,25,27-29]. Moreover, *DJ-1−/−* dopaminergic neurons or siRNA-mediated knockdown of DJ-1 mRNA in primary embryonic midbrain DA neurons resulted in increased sensitivity to toxins that induce oxidative stress [30]. DJ-1 is also abundantly expressed in non-neuronal cells and the bacterial endotoxin lipopolysaccharide (LPS) induces a robust increase in DJ-1 expression in inflammatory cells such as peritoneal macrophages [31]. LPS exposure causes astrocytes derived from *DJ-1−/−* mice to generate ten times more nitric oxide than astrocytes derived from wild-type mice [32]. These studies suggest that DJ-1 loss-of-function mutations affect both neuronal and non-neuronal cell types and could result in enhanced microglial activation upon neuroinflammatory insults. Therefore, the purpose of our study was to investigate whether loss of DJ-1 protein increases the vulnerability for inflammation-induced nigrostriatal degeneration *in vivo*. To this end, we investigated the extent to which repeated intranasal administration of soluble tumor necrosis factor (inTNF) or repeated intraperitoneal (i.p) injections of low-dose LPS might induce neuroinflammation, locomotor deficits, enhance oxidative stress and/or elicit nigral DA neuron loss in *DJ-1−/−* mice compared to wild-type mice.

## Methods

### Animals

*DJ-1−/−* mice were generated and characterized as described previously [17]. Prior to these studies, *DJ-1−/−* mice were back-crossed onto a C57BL/6 genetic background for over ten generations. Mice were housed in a pathogen-free, climate controlled facility in the Animal Resources Center at The University of Texas Southwestern Medical Center at Dallas and given food and water *ad libitum*. All animal studies were reviewed and approved by the Institutional Animal Care and Use Committee at The University of Texas Southwestern Medical Center in accordance with the National Institutes of Health Guide for the Care and Use of Laboratory Animals.

### Intranasal tumor necrosis factor administration

Murine soluble TNF (0.5 ng or 5 ng delivered as 5 μL of 0.1 ng/uL or 5 μL of 1 ng/uL) or an equivalent amount of saline vehicle was administered intranasally via an L-10 Pipetman (Rainin Instrument, Oakland, CA, USA) twice weekly for the time indicated in each set of experiments. Wild-type or *DJ-1−/−* mice were 12 months old at the start of the study (Additional file 1: Figure S1A) and 15 months old at the time of sacrifice.

### Systemic lipopolysaccharide administration

The regimen of LPS injections was chosen based on our previous work which demonstrated that this dose and frequency of i.p. LPS triggers a neuroinflammatory response in the midbrain and elicits nigral DA neuron loss in *parkin*−/− mice [33]. Young adult (6 to 13 week old) *DJ-1−/−* mice on a C57BL/6 background and age-matched wild-type mice were given either 7.5 × 10^5^ EU/kg LPS from *Escherichia coli* O111:B4 (Sigma-Aldrich, Saint Louis, MO, USA) or sterile 0.9% sodium chloride vehicle control (Braun Medical, Inc., Irvine, CA, USA) i.p. injections twice a week for 3 months. A second group of mice (designated as 3-month/3-month wait) was given systemic LPS or vehicle i.p. injections for 3 months followed by a 3-month wait period prior to tissue collection, during which no additional i.p. injections were administered (n = 3 to 6 per group). A third group of mice was given twice weekly systemic LPS or vehicle injections for 6 months with no wait period before tissue collection (n = 3 to 7 per group). We would like to note that the *DJ-1−/−* versus wild-type mouse studies reported in this manuscript were performed alongside a cohort of *parkin−/−* mice, the results of which were reported previously [33] in comparison to the same cohort of wild-type mice used here.

### Behavior testing

For all behavioral tests, mice were evaluated at baseline (before i.p. injections began) and again after 3 or 6 months of treatment.

#### Open-field

Open-field behavior in a glass container (diameter, 24.5 cm) was recorded for 5 minutes for evaluation of time spent moving and number of rearing events by an investigator blinded to genotype and treatment history.

#### Narrow beam walk

A narrow beam (1.1 cm diameter, 80.6 cm testing length) with a home cage at one end was used. Initial training prior to treatment consisted of three sessions of three trials per session for 4 consecutive days. Mice received additional training sessions at 3 months and 6 months after the start of the treatment regimen consisting of three sessions of three trials per session on 1 day. Testing was conducted the day after training and consisted of one session of three trials. The average time to traverse the full length of the beam was used for data analysis.

#### Accelerating rotarod

A base speed of 20 rpm with an acceleration of 0.2 rpm/second was used on the rotarod (Economex 0207–005 M, Columbus Instruments, Columbus, OH, USA). Mice were trained prior to treatment in three sessions of four trials each for 4 consecutive days. Mice received additional training sessions at 3 months and 6 months after the start of treatment consisting of three sessions of four trials per session on 1 day. Testing consisted of one session of three trials the day after training was completed. Latency to fall (seconds) was calculated and used for data analysis.

### Tissue harvest

Following the last inTNF administration or i.p. injection and final behavioral testing, mice in the 3-month, 3-month/3-month wait, and 6-month treatment cohorts were deeply anesthetized with Euthasol (pentobarbital sodium and phenytoin sodium) i.p. then intracardially perfused with warm 0.1 M PBS pH 7.4 supplemented with 0.1% glucose and 1 U/mL heparin prior to rapid whole brain removal. For quantitative real-time PCR (QPCR), brain tissue was microdissected into four regions on an ice-cold glass Petri dish - olfactory bulb, cerebellum, ventral midbrain and cortex - then snap-frozen in cryovials in liquid nitrogen and stored at −80°C until processed for RNA extraction. For immunohistochemistry, mice in the 3-month, 3-month/3-month wait, and 6-month treatment cohorts were perfused with warm 0.1M PBS (pH 7.4 supplemented with 0.1% glucose and 1 U/mL heparin) followed by ice cold 4% paraformaldehyde in PBS (pH 7.4). Brains (in the skull) were post-fixed overnight in 4% paraformaldehyde. Brains were removed from the skull and then were cryoprotected for 24 hours in 20% sucrose in 0.1 M PBS pH 7.4, embedded in Neg 50 frozen section medium (Richard Allen Scientific, Kalamazoo, MI, USA), and frozen in dry ice-cooled isopentane.

### Peritoneal macrophage harvest

Murine peritoneal macrophages were obtained by eliciting an acute peripheral inflammatory reaction with an i.p. injection of thioglycolate [34]. Briefly, adult mice were given an i.p. injection of 3% Brewer’s yeast thioglycolate in normal saline. Three days later, animals were euthanized and peritoneal exudates were recovered, pelleted and resuspended in culture media (high-glucose DMEM supplemented with 10% heat-inactivated FBS from Atlanta Biologicals (Norcross, GA, USA), 1% penicillin/streptomycin, and 1% L-glutamine (Sigma-Aldrich)). Six hours after the initial plating, cells were washed twice with PBS without Ca^2+^ or Mg^2+^ to remove non-adherent cells and growth medium was replenished to the homogeneous population of adherent macrophages.

### Quantitative real-time polymerase chain reaction

Total RNA was isolated from tissue samples using Tri Reagent® (Molecular Research Center, Cincinnati, OH, USA), treated with DNAse I (Invitrogen, Carlsbad, CA, USA), and reverse transcribed to obtain cDNA. QPCR was performed using SYBR Green Master Mix (ABI) on an Applied Biosystems Prism 7900HT sequence detection system (Foster City, CA, USA) as described previously [35]. Primers for each gene (available upon request) were designed using Primer Express Software (PerkinElmer Life Sciences, Wellesley, MA, USA) and were validated by analysis of template titration and dissociation curves. Results for QPCR were normalized to the housekeeping gene cyclophilin B and evaluated by comparative C_T_ method (user bulletin No. 2, PerkinElmer Life Sciences). RNA levels are expressed relative to the wild-type saline-injected (vehicle) mice.

### Immunohistochemistry

Coronal serial sections (30 μm thickness) were cut on a Leica CM 1850 cryostat (Buffalo Grove, IL, USA) and placed on Superfrost/Plus microscope slides (Fisher Scientific; Pittsburgh, PA, USA). Sections on slides were stored at −80°C until processed for immunohistochemistry.

*Brightfield immunohistochemistry*. Sections were stained for tyrosine hydroxylase (TH) using published protocols [36,37]. Sections were permeabilized in 0.3% Triton X-100 in PBS pH 7.4. Endogenous peroxidases were quenched with 1% H_2_O_2_ and non-specific binding was blocked with 5% normal serum (goat or horse, Equitech-Bio, Inc., Kerrville, TX, USA). Sections were incubated with primary antibodies against TH (rabbit polyclonal antibody AB152 diluted 1:2000, Chemicon International, Temecula, CA, USA), or neuronal nuclear antigen (NeuN) (mouse monoclonal antibody MAB377 diluted 1:1000, Chemicon) overnight at room temperature followed by biotinylated secondary antibody (goat anti-rabbit or horse anti-mouse rat absorbed, or goat anti-rat IgG diluted 1:400, Vector Laboratories, Burlingame, CA, USA) and NeutrAvidin-HRP (diluted 1:5000, Pierce Biotechnology, Inc., Rockford, IL, USA). The tissue bound peroxidase activity was developed with 0.024% diaminobenzadine (DAB, Sigma), 0.006% H_2_O_2_ in 0.05 M Tris–HCl buffer pH 7.6 for 20 minutes with or without nickel intensification. Tissue sections were dehydrated in a graded series of ethanols, immersed in xylene, and coverslipped with Permount (Fisher Scientific).

*Fluorescence immunohistochemistry for Iba1-positive microglia*. Brain sections were stained for microglial markers using a standard immunofluorescence protocol [12]. Auto-fluorescence was quenched in 0.2 M glycine in PBS pH 7.4, for 1 hour at room temperature. Sections were then permeabilized in 0.3% Triton X-100 with 1% normal goat serum in 20 mM Tris-buffered saline (TBS) pH 7.4. Non-specific binding was blocked with species-appropriate 1% normal serum in TBS. Sections were incubated overnight at 4°C with an antibody specific for Iba1 (Wako Chemicals, Richmond, VA, USA, 019–19741, diluted 1:10000) and followed by Alexa-conjugated secondary antibody (Fab) (1:1000 dilution, Invitrogen) for 4 hr at room temperature. Antibodies were diluted in blocking buffer with 0.1% Triton X-100. Washes were done in TBS with 0.2% Triton X-100 (TBST). Following secondary antibody incubations, the slides were rinsed briefly with dH_2_O, then counterstained with Hoescht 33258 (diluted 1:20,000, Invitrogen) for 15 minutes, and coverslipped with aqueous mounting media with anti-fade (Biomeda Corp, Foster City, CA, USA). Quantification of Iba1-positive cells was performed on images captured under a 20X objective lens (or 40X for inset images) on a Nikon 90i fluorescence microscope using thresholding analysis on Nikon Elements 5 software (Nikon Instruments, Melville, NY, USA). Values represent the mean ± SEM of Iba1-positive microglia per field from four fields selected randomly from entorhinal brain sections harvested from two mice per treatment group.

### Stereological analysis

The optical fractionator probe of Stereoinvestigator software (MicroBrightField, Inc., Williston, VT, USA) was used to obtain an unbiased estimate of TH-positive and NeuN-positive neurons in the SNpc and ventral tegmental area (VTA) as per the atlas of the mouse brain by Paxinos and Franklin [38]. Stereologic parameters were as follows: counting frame, 50 μm × 50 μm; optical dissector: 20 μm; grid size, 120 μm × 160 μm. For the population size estimate (number of sections per animal), a target coefficient of error (Gundersen’s m = 1) of less than 0.10 was considered acceptable. Neuron counting was performed by two different investigators blinded to genotype and treatment history.

### Striatal tyrosine hydroxylase fiber density and densitometry

Coronal serial sections (30 μm thickness) were cut between Bregma −1.22 to 1.70 on a Leica CM 1850 cryostat and placed on Superfrost/Plus microscope slides (Fisher Scientific). Tissue sections were immunostained for TH and developed using DAB as described above. Images of striatum (caudate putamen) from 12 tissue sections per animal were taken with a CoolSnap cf digital color camera mounted on a BX61 microscope (Olympus, Center Valley, PA, USA). Exposure times were kept constant for all images. TH-positive fiber density was determined using background corrected integrated optical density measurements for each section using an Alpha Innotech FluorChem FC2 imaging workstation and software (Protein Simple, Santa Clara, CA, USA). All sections for each animal were averaged and group means were used to compare treatment groups.

### Striatal dopamine and metabolites

Levels of striatal DA and its metabolites (DOPAC, HVA and 3-MT) were quantified by HPLC with electrochemical detection. Mice were euthanized by carbon dioxide asphyxiation followed by immediate decapitation and dissection of the striatum on an ice-cold glass Petri dish. The striatum was then weighed and stored at −80°C. Frozen brain tissue was sonicated in a 49 volume/weight (mg of tissue) solution of 0.1 M perchloric acid containing 0.2 mM sodium metabisulfite and centrifuged at 20,000 rpm for 20 minutes at 4°C in a benchtop centrifuge to clear debris. Cleared supernatant (20 μL) was then injected onto a C18 HPLC column (ESA MD-150 3 × 150 mm) and separated by isocratic elution at a flow rate of 0.6 ml/minute using MD-TM mobile phase (ESA Inc., Chelmsford, MA, USA). Neurotransmitter monoamines and metabolites were detected using a BAS electrochemical cell set to a potential of +800 mV and compared to external standards. Dopamine turnover was calculated as (DOPAC + HVA + 3-MT)/DA.

### Statistics

Multiple-way analysis of variance (ANOVA) with significance level α = 0.05 were used as indicated for each set of experimental data. Significant differences between groups were further evaluated using Tukey’s HSD or Bonferroni’s *post hoc* test. Kruskal-Wallis analysis was the non-parametric statistical test used for testing equality of population medians of integrated optical density measurements of striatal TH fiber density. Graphs were generated and statistical analyses performed with the use of GraphPad Prism 5.0 (GraphPad Software, La Jolla, CA, USA).

## Results

### Intranasal soluble tumor necrosis factor delivery does not promote nigral dopamine neuron loss in wild-type or *DJ-1−/−* mice

Neuroinflammation can enhance oxidative stress responses and, based on evidence that DJ-1 plays a protective role against oxidative stress, we investigated whether *DJ-1−/−* mice display increased vulnerability to inflammation-induced nigral degeneration. First, we tested an inflammatory regimen designed to trigger neuroinflammation directly in the olfactory bulb, a brain region that has been demonstrated to show early involvement in PD pathogenesis [39]. We delivered rhodamine-labeled human TNF (rh-TNF) intranasally to confirm uptake and transport by nasal epithelia (NE). Immunofluorescence analyses of cryosectioned rostral and caudal NE tissues revealed detectable uptake and transport of rh-TNF in rostral sections of NE at early time points (data not shown). As expected, the distribution of rh-TNF progressed from rostral to caudal sections as a function of time (over a 12-hour period). Because TNF can induce cachexia, we investigated the extent to which inTNF administration decreased food and water intake. Soluble recombinant mouse TNF was administered intranasally twice weekly at one of two doses (0.5 ng or 5 ng) for 1 week or for 2 weeks in wild-type and *DJ-1−/−* mice. Food consumption and body weight were monitored just prior to and during the inTNF dosing. Results revealed no adverse systemic effects (that is, loss of appetite or body weight) after repeated systemic inTNF dosing (data not shown). Next, we investigated whether inTNF administration in wild-type or *DJ-1−/−* mice resulted in detectable nigral DA neuron loss. We subjected 12-month old *DJ-1−/−* mice and wild-type mice to an inTNF experimental regimen that consisted of four initial administrations of soluble TNF at the lower dose (0.5 ng, chosen to minimize potentially adverse systemic effects from repeated administration) followed by several boost doses (at 0.5 ng) and a 2-month wait time with the intent to trigger an inflammatory response that might persist or progress beyond the initial inflammatory stimulus (Additional file 1: Figure S1A). Stereological analyses to determine the extent to which the experimental paradigm induced nigral DA neuron loss revealed no inTNF-induced nigral DA neuron loss in mice of either genotype (Additional file 1: Figure S1B). Consistent with our immunohistological findings, measurements of striatal DA and DA turnover by HPLC with electrochemical detection showed no inTNF-induced neurotransmitter changes in wild-type or *DJ-1−/−* mice (Additional file 1: Figure S1C). Compared to other groups, we observed a significant reduction in striatal DA in vehicle-treated *DJ-1−/−* mice, but inTNF administration did not further decrease striatal DA or affect DA turnover (Additional file 1: Figure S1C).

**Figure 1:**
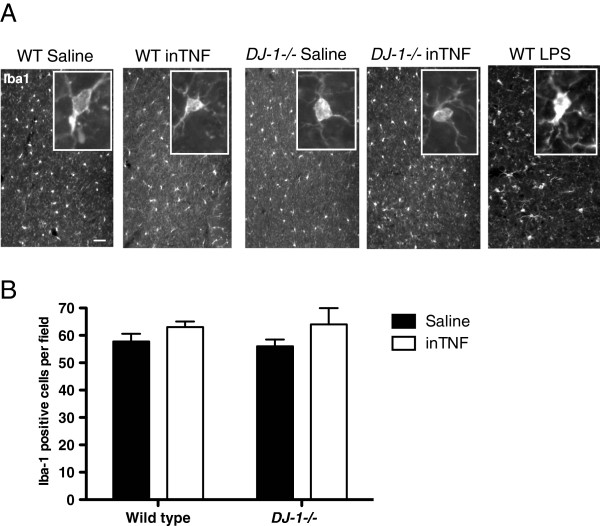
**Intranasal soluble tumor necrosis factor dosing regimen does not induce robust microglia activation in the midbrain.** (**A**) Immunohistochemical analysis for the microglia marker Iba1 in representative brain sections from cortical regions of wild-type (WT) or *DJ-1−/−* mice exposed to repeated intranasal administration of saline or soluble TNF (inTNF) (0.5 ng) as indicated in the experimental paradigm (Additional file 1: Figure S1A), or to 3-month intraperitoneal lipopolysaccharide (LPS) (See Methods). Scale bar = 200μm. (**B**) Quantification of Iba1-positive cells within the entorhinal cortex of inTNF-treated WT or *DJ-1−/−* mice. Analysis by two-way analysis of variance revealed no gross differences due to genotype or treatment in microglia morphology or number of Iba1-positive microglia per field (n = 4 fields per treatment group).

We considered that the failure of the inTNF regimen to induce nigral DA neuron degeneration could be due to a limited neuroinflammatory response. Therefore, we investigated the extent to which the repeated inTNF dosing (Additional file 1: Figure S1) triggered neuroinflammation in wild-type and *DJ-1−/−* mice. We found that inTNF exposure did not result in significant changes in microglia morphology (Figure 1A) or increases in microglia number in either wild-type or *DJ-1−/−* mice as quantified by Iba1 immunohistochemistry in cortical sections (Figure 1B). As a positive control for microglial activation, we included Iba1-positive microglia in wild-type mice treated with i.p. LPS for 3 months (Figure 1A). Therefore, because the inTNF regimen failed to trigger a robust inflammatory response in the midbrain, we opted to use a different regimen to trigger neuroinflammation in the midbrain that would allow us to investigate the extent to which loss of DJ-1 protein might predispose mice to inflammation-induced nigral degeneration. The regimen consisted of repeated i.p. LPS injections previously shown to induce equivalent neuroinflammatory responses in both wild-type and *parkin−/−* mice, but significant nigral DA neuron loss only in *parkin−/−* mice [33].

### *DJ-1−/−* mice exposed to prolonged, serial low-dose systemic lipopolysaccharide do not develop locomotor deficits

**Figure 2:**
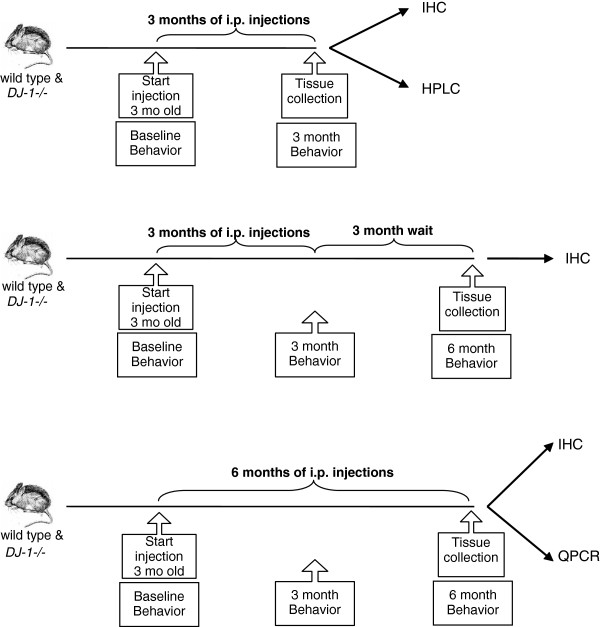
**Schematic of systemic lipopolysaccharide administration regimens and measurable outcomes.** Wild-type and *DJ-1−/−* mice were given low-dose lipopolysaccharide or an equivalent volume of saline vehicle twice a week intraperitoneally (i.p.) for the indicated times. Locomotor behavior was evaluated before and during the course of treatment. Groups of mice were sacrificed as indicated for biochemical and immunohistological analyses. IHC, immunohistochemistry; mo, months; QPCR, quantitative polymerase chain reaction; HPLC, high-performance liquid chromatography.

**Figure 3:**
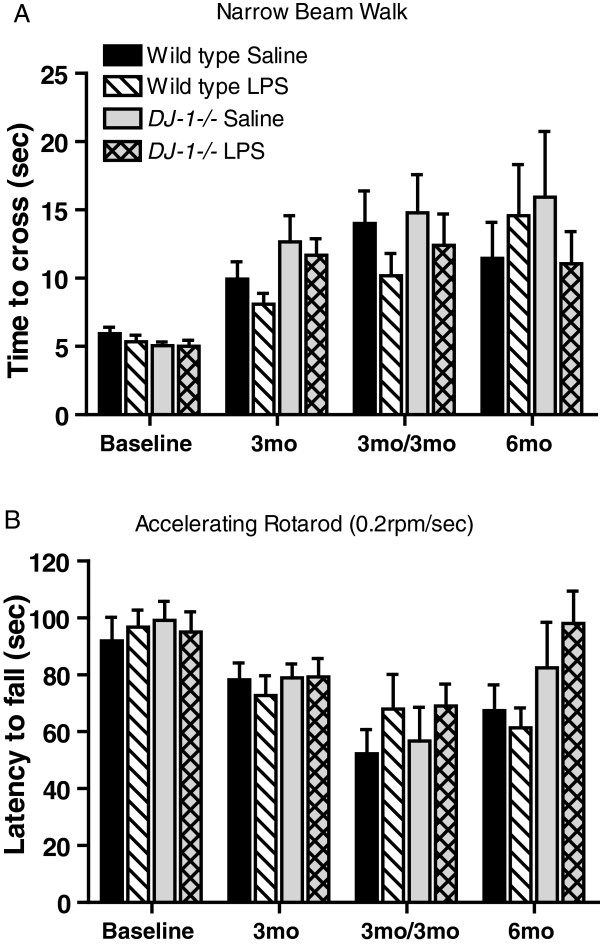
**Absence of motor deficits in *DJ-1−/− *mice exposed to prolonged, serial low-dose systemic lipopolysaccharide.** No significant differences were detected between genotypes or treatment groups in (**A**) the time to walk across a narrow beam or (**B**) the time before falling off an accelerating rotarod, suggesting no general malaise or overt motor deficits compared to wild-type saline-treated mice. Bars represent mean ± SEM; n = 8 per group. Analysis by three-way analysis of variance followed by a Tukey’s HSD *post hoc* test at *P* < 0.05 for significance. For rotarod: genotype F_(1, 232)_ = 3.46, *P* = 0.064; treatment F_(1, 232)_ = 0.76, *P* = 0.383. For beam walk: genotype F_(1, 232)_ = 1.86, *P* = 0.173; treatment F_(1, 232)_ = 2,92, *P* = 0.089. LPS, lipopolysaccharide; mo, months.

To test the hypothesis that DJ-1 regulates vulnerability to inflammation-related nigral degeneration, we exposed wild-type and *DJ-1−/−* mice to various systemic LPS regimens (Figure 2). A number of behavioral tasks were measured at the time points indicated to investigate the extent to which the prolonged serial low-dose systemic LPS regimens induced locomotor alterations. To assess fine-motor performance, mice were subjected to the narrow beam-walk test while gross motor abilities were measured by rotarod and open field performance. Our results indicate that *DJ-1−/−* mice chronically injected with LPS did not display significantly slower average time-to-cross on the narrow beam-walk test (Figure 3A), nor did they exhibit reduced performance on the rotarod (Figure 3B) or altered open field behavior (data not shown) compared to saline-treated *DJ-1−/−* mice or wild-type mice of either treatment group. Thus, in the present study, prolonged serial i.p. injections of LPS or saline did not cause gross or fine motor abnormalities.

### Chronic low-dose systemic lipopolysaccharide does not promote loss of nigral dopamine neurons or striatal dopaminergic terminals in wild-type or *DJ-1−/−* mice

**Figure 4:**
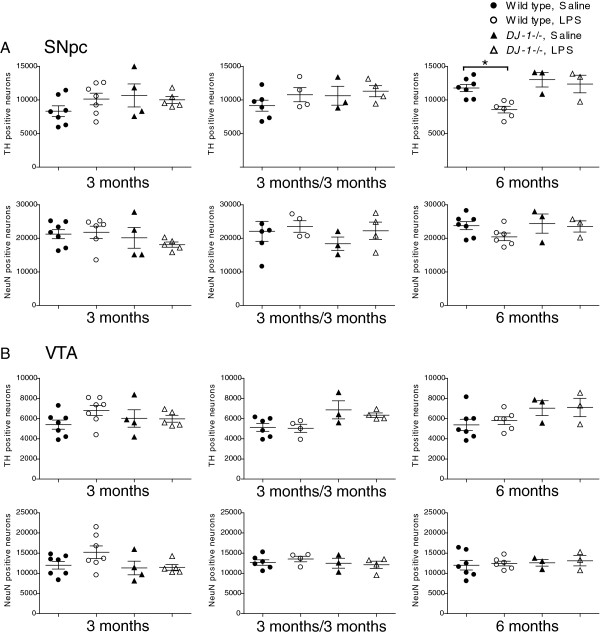
***DJ-1−/− *mice do not display increased vulnerability to nigral dopamine neuron loss induced by repeated low-dose systemic lipopolysaccharide compared to wild-type mice.** Unbiased stereological analysis indicates that *DJ-1−/−* mice exposed to 3 or 6 months of low-dose systemic lipopolysaccharide (LPS) or to 3 months of low dose systemic LPS followed by a 3 month wait before analysis do not display a significant reduction of tyrosine hydroxylase (TH) or NeuN immunopositive neurons in (**A**) the substantia nigra pars compacta (SNpc) or (**B**) the ventral tegmental area (VTA). Error bars represent SEM; analysis includes n = 3 to 7 per group in final cohorts due to attrition. Asterisks indicate significant differences compared with wild-type, saline-treated mice by three-way analysis of variance followed by Tukey’s HSD *post hoc* test at *P* < 0.05. For SNpc TH neurons: genotype F_(1, 47)_ = 7.89, *P* = 0.007; treatment F_(1, 47)_ = 0.01, *P* = 0.904. For SNpc NeuN neurons: genotype F_(1, 47)_ = 1.05, *P* = 0.311; treatment F_(1, 47)_ = 0.0.03, *P* = 0.858. For VTA TH neurons: genotype F_(1, 47)_ = 8.57, *P* = 0.005, *post hoc* analysis reveal no significant differences; treatment F_(1, 47)_ = 0.39, *P* = 0.536. For VTA NeuN neurons: genotype F_(1, 47)_ = 1.26, *P* = 0.268; treatment F_(1, 47)_ = 01.28, *P* = 0.269.

To estimate the number of DA neurons and total neurons in the SNpc, we performed immunohistochemistry for TH as the dopaminergic neuronal marker and NeuN as the pan-neuronal marker. Compared to similarly dosed wild-type mice, *DJ-1−/−* mice that received 3 months of repeated low-dose systemic LPS did not exhibit significant reductions in TH positive or NeuN positive neurons in the SNpc (Figure 4A). Similarly, 3 months of repeated low-dose systemic LPS followed by a 3-month wait period did not significantly reduce the number of TH positive or NeuN positive SNpc neurons in *DJ-1−/−* mice or wild-type mice (Figure 4A). An ANOVA followed by a Tukey’s HSD *post hoc* test indicated a significant (~29%) decrease in TH-positive nigral neurons in wild-type mice treated with LPS for 6 months, compared to saline-treated wild-type mice; however, no significant difference was found in the number of NeuN-positive nigral neurons (~13% decrease in LPS-treated compared to saline-treated wild-type mice), suggesting that these results may reflect downregulation of TH expression (Figure 4A). The numbers of TH- and NeuN-positive neurons were also unchanged in the VTA (Figure 4B).

**Figure 5:**
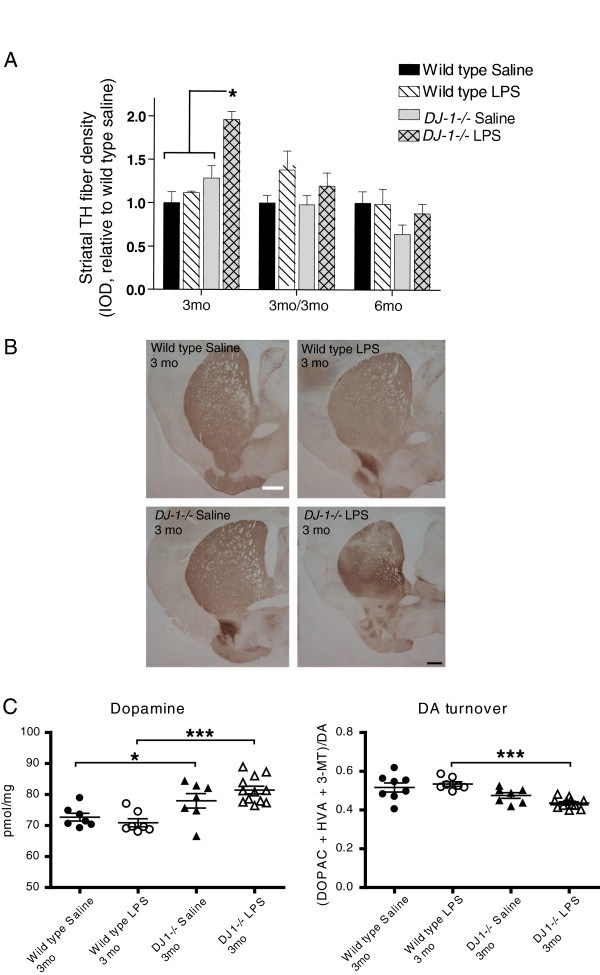
**Repeated low-dose intraperitoneal lipopolysaccharide injections do not induce loss of striatal tyrosine hydroxylase-immunopositive terminals or dopamine depletion in *DJ-1−/− *or wild-type mice. **(**A**) Densitometric analysis of striatal tyrosine hydroxylase (TH) fiber density indicates no significant differences between genotypes or treatment groups as indicated by Kruskal-Wallis with Tukey’s *post hoc* test. Bars represent mean ± SEM; analysis includes n = 3 to 7 per group in final cohorts due to attrition. The asterisk represents difference between bracketed groups and *DJ-1−/−* LPS. (**B**) Representative striatal sections stained for TH from mice in the 3 month treatment groups; scale bar = 400μm. (**C**) Striatal levels of dopamine (DA) were measured by HPLC electrochemical detection for mice in the 3-month treatment group and striatal DA turnover was calculated as the ratio of DA metabolites (DOPAC, HVA and 3-MT) to DA. Asterisks indicate significant differences compared with wild-type animals by two-way analysis of variance followed by Bonferroni’s HSD *post hoc* test at *P < 0.05 and ***P < 0.001 (Genotype effect for DA: F_(1,30)_ = 26.01, *P* < 0.001; DA turnover: F_(1,29)_ = 23.49, *P* < 0.001). IOD, integrated optical density; LPS, lipopolysaccharide; mo, months; DA, dopamine.

In PD, loss of dopaminergic nerve terminals in the striatum presumably precedes loss of cell bodies within the SNpc. Therefore, to investigate whether repeated low-dose systemic LPS affected dopaminergic terminals in the striatum, we analyzed striatal sections using TH immunohistochemistry. No decreases in the density of TH-positive fibers were detectable in striatal brain regions of *DJ-1−/−* mice treated with low-dose systemic LPS for 3 or 6 months or for 3 months followed by a 3-month wait period (Figure 5B). Densitometric analysis of multiple sections indicated that the only significant change occurred in *DJ-1−/−* mice exposed to LPS for 3 months; these mice displayed an increase in striatal TH-positive fiber density (Figure 5A). To extend and confirm these findings, we measured the tissue levels of DA and its metabolites in microdissected striatum by HPLC with electrochemical detection. In agreement with the immunohistochemical results, repeated low-dose i.p. LPS injections caused a detectable increase in striatal DA content in *DJ-1−/−* mice exposed to the 3-month regimen (Figure 5C). The increase in striatal DA in this group is accompanied by a slight decrease in DA turnover, which is calculated as the ratio of DA metabolites (DOPAC, HVA and 3-MT) to DA. There were no significant differences in levels of DOPAC, HVA or 3-MT levels (Additional file 2: Figure S2).

### Low-dose systemic lipopolysaccharide administration causes neuroinflammatory responses in midbrains of *DJ-1−/−* and wild-type mice

**Figure 6:**
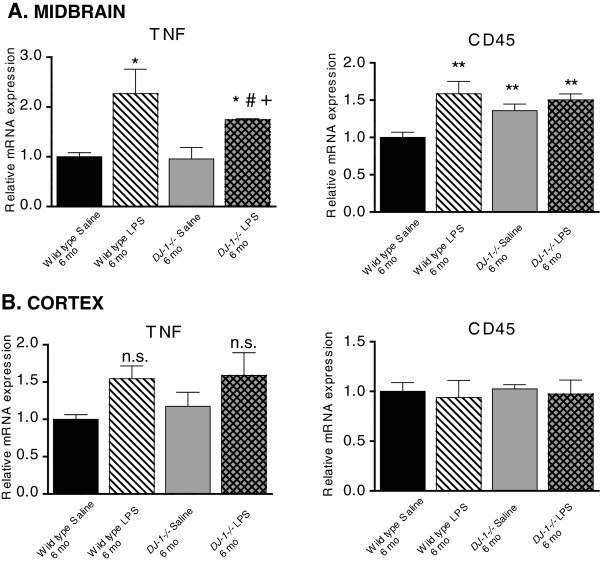
***DJ-1−/− *display blunted neuroinflammatory responses in midbrain compared to wild-type mice after repeated low-dose systemic lipopolysaccharide administration.** Quantitative PCR analyses of microdissected brain tissue was used to measure expression levels of the neuroinflammation markers TNF and CD45 in (**A**) the ventral midbrain and (**B**) the cortex of mice treated with low-dose systemic lipopolysaccharide (LPS) (or saline) for 6 months (mo). Parkin mRNA was also measured and found to be similar in both genotypes (data not shown). *,**Significant difference from wild-type saline; #Significant difference from *DJ-1−/−* saline; +Significant difference between LPS conditions. Bars represent mean ± SEM; n = 3 to 4 per group. A two-way analysis of variance was performed with Tukey’s HSD *post hoc* at *P* < 0.05 (midbrain TNF treatment effect: F_(1,9)_ = 16.57, *P* = 0.0028, genotype effect *P* = 0.29; midbrain CD45 treatment effect: F_(1,10)_ = 9.38, *P* = 0.0120, genotype effect *P* = 0.09; cortex TNF treatment effect: F_(1,10)_ = 6.73, *P* = 0.0268, genotype effect *P* = 0.73; cortex CD45: not significant (n.s.), treatment *P* = 0.679, genotype *P* = 0.97).

To investigate the possibility that the lack of nigral neuron loss in LPS-treated *DJ-1−/−* mice could be attributed to an attenuated neuroinflammatory response, we used QPCR to measure the relative mRNA expression of TNF and CD45 (a marker of activated monocytes also known as leukocyte common antigen) in the midbrain and cortex of the mice from the 6-month treatment groups. We found that basal levels of TNF mRNA were similar in wild-type and *DJ-1−/−* mice within the midbrain and cortex of saline-treated animals, and i.p. LPS treatment evoked increases in TNF mRNA in the midbrain (but not cortex) of wild-type and *DJ-1−/−* mice (Figure 6A,B). Surprisingly, the level of TNF mRNA was higher in the midbrains of LPS-treated wild-type mice compared to LPS-treated *DJ-1−/−* mice (Figure 6A). Interestingly, the basal levels of midbrain (but not cortex) CD45 mRNA were elevated in *DJ-1−/−* mice relative to wild-type mice, and i.p. LPS treatment evoked increased CD45 mRNA in the midbrain of wild-type mice but no further increases were observed in *DJ-1−/−* mice over basal levels.

### *DJ-1−/−* mice display blunted anti-oxidant gene responses relative to wild-type mice after exposure to repeated low-dose systemic lipopolysaccharide administration

**Figure 7:**
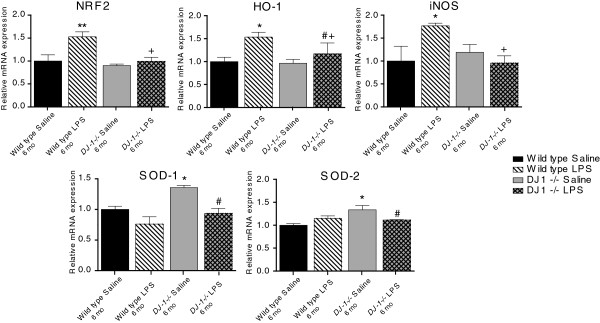
***DJ-1−/− *mice display blunted oxidative stress responses to prolonged, serial administration of low-dose systemic lipopolysaccharide relative to wild-type mice.** Real-time quantitative PCR analyses of microdissected midbrain tissue from mice treated with low-dose lipopolysaccharide (LPS) (or saline) for 6 months (mo) measured expression levels of NF-E2 related factor (Nrf2), heme-oxygenase-1 (HO-1), inducible nitric oxide synthase (iNOS), superoxide dismutase-1 (SOD-1), and superoxide dismutase-2 (SOD-2). *,**Significant difference from wild-type saline; #Significant difference from *DJ-1−/−* saline; +Significant difference between the LPS conditions. Bars represent mean ± SEM; n = 3 to 4 per group. A two-way analysis of variance was performed with Tukey’s HSD *post hoc* at *P < 0.05 (Nrf2 treatment effect: F_(1,10)_ = 7.96, *P* = 0.0181, genotype effect: F_(1,10)_ = 8.28, *P =* 0.016; HO-1 treatment effect: F_(1,10)_ = 7.56, *P* = 0.0205, genotype effect: *P =* 0*.*166; iNOS not significant: treatment effect, *P* = 0.2474, genotype effect, *P* = 0.183; however, a significant relationship between treatments and genotype did exist (F_(1,10)_ = 5.318, *P* = 0.044); SOD-1 treatment effect: F_(1,10)_ = 14.67, *P* = 0.0033, genotype effect: F_(1,10)_ = 9.47, *P* = 0.012 SOD-2 treatment effect: not significant, *P* = 0.5175, genotype effect: F_(1,10)_ = 7.08, *P* = 0.024).

We next investigated whether the lack of nigral cell loss in *DJ-1−/−* mice following repeated, serial systemic low-dose LPS administration could be attributed to upregulation of anti-oxidant responses as a developmental consequence of DJ-1 deficiency. We used QPCR to measure the expression of key anti-oxidant genes implicated in protection of DA neurons in the midbrain in wild-type or *DJ-1−/−* mice that received a 6-month low-dose systemic LPS administration or saline control. We analyzed mRNA levels of the transcription factor NF-E2 related factor (Nrf2), which binds to the anti-oxidant response element to induce expression of anti-oxidant and phase 2 detoxification enzymes, heme-oxygenase-1 (HO-1), inducible nitric oxide synthase (iNOS), superoxide dismutase-1 (SOD-1), and superoxide dismutase-2 (SOD-2). We found upregulation of SOD-1 and SOD-2 mRNA in the midbrain of saline-treated *DJ-1−/−* mice compared to wild-type saline animals (Figure 7). In contrast to wild-type mice, low-dose peripheral LPS treatment in *DJ-1−/−* mice resulted in a significant reduction in SOD-1 and in SOD-2 mRNA relative to saline treatment. Low-dose peripheral LPS treatment evoked significant increases in Nrf2, iNOS, and HO-1 mRNA in the wild-type mice midbrain but failed to elicit a similar response in *DJ-1−/−* mice. Although HO-1 mRNA was significantly increased in midbrains of LPS-treated *DJ-1−/−* mice compared to saline-treated *DJ-1−/−* mice, it was significantly less than in LPS-treated wild-type mice. Taken together, LPS treatment did not elicit an upregulation of anti-oxidant genes in *DJ-1−/−* mice compared to wild-type mice, indicating a blunted response in *DJ-1−/−* midbrains. Therefore, the lack of inflammation-induced nigral degeneration was not due to an enhanced anti-oxidant response in *DJ-1−/−* mice.

### *DJ-1−/−* and wild-type macrophages display similar inflammatory and anti-oxidant gene responses

To determine whether the blunted oxidative stress response in the midbrain after chronic peripheral inflammation was due to *DJ-1* gene ablation in cell types that typically respond to LPS with inflammatory and oxidative bursts, we isolated peritoneal macrophages from wild-type or *DJ-1−/−* mice and tested their responses to LPS *in vitro*. QPCR analyses revealed that DJ-1 mRNA levels are comparable in microglia and peritoneal macrophages (data not shown). Gene expression analyses of TNF, iNOS, IL-1β, Nrf2, and NQO1 mRNA from isolated macrophages revealed no difference between genotypes (Additional file 3: Figure S3). Lastly, our previous work found that Parkin function may influence neuroinflammatory responses and vulnerability to inflammation-induced nigral degeneration [33], and we considered that perhaps Parkin levels were compensatorily upregulated in *DJ-1−/−* mice, and contribute to the lack of vulnerability of *DJ-1−/−* mice to inflammation-induced nigral degeneration. However, we found no differences between genotypes or treatment groups in the levels of Parkin mRNA or protein expression in the midbrain and cortex or in peritoneal macrophages (data not shown).

## Discussion

DJ-1 function has been implicated in the regulation of inflammation-induced oxidative stress *in vitro* by reports that LPS can robustly increase DJ-1 expression in peritoneal macrophages in response to NADPH oxidase-derived reactive oxygen species [31]. Moreover, DJ-1 has been shown to be important for mitochondrial function in astrocytes [40] and *DJ-1−/−* astrocytes overproduce nitric oxide when stimulated with LPS [32,41]. On the basis of these *in vitro* studies we expected that the brains of *DJ-1−/−* mice would display exacerbated neuroinflammatory responses to chronic inflammatory stress and would develop a nigral degeneration phenotype, given that DA neurons are sensitive to neuroinflammation. However, we found that the neuroinflammatory responses in *DJ-1−/−* mice after repeated i.p. LPS injection were similar to that of wild-type mice (Figure 6). Chronic i.p. LPS exposure induced significant increases in midbrain TNF levels in both wild-type and *DJ-1−/−* mice; however, the TNF levels were higher in LPS-treated wild-type mice compared to LPS-treated *DJ-1−/−* mice. It remains possible that *DJ-1−/−* mice may have shown significant loss of nigral dopamine neurons if the LPS dose were increased to cause midbrain TNF levels to match that of wild-type mice treated with this dose of LPS. It is worth noting that the microglia burden measured by Iba-1+ cells in the brains of *DJ-1−/−* mice was not different from wild-type mice (Figure 1), and is consistent with the observation that the lineage-related myeloid-derived peripheral macrophage population also displayed inflammatory responses *in vitro* that were indistinguishable from wild-type (Additional file 2: Figure S3). inTNF administration caused no apparent neuroinflammatory responses in either wild-type or *DJ-1−/−* mice. Therefore, the magnitude of the TNF inflammatory insult may be too low to draw conclusions regarding the effect of DJ-1 mutations on susceptibility to TNF neuroinflammation-mediated neurodegeneration. It remains possible that we would have observed different responses to TNF in wild-type and *DJ-1−/−* mice if we had used a stronger TNF administration regimen.

Functionally, the repeated i.p. LPS injection regimen which successfully triggered nigral degeneration in *parkin−/−* mice [33] did not induce nigral degeneration in *DJ-1−/−* mice (Figure 4), suggesting that loss of DJ-1 function does not increase vulnerability to inflammation-induced nigral degeneration. Nevertheless, it remains possible that a higher concentration of inTNF or LPS and/or a longer regimen (greater than 6 months) and/or longer wait-time (>3 months) between the delivery of the last set of repeated i.p. LPS injections and the endpoint of the study may be required to elicit a more robust neuroinflammatory response and uncover increased vulnerability to inflammation-induced degeneration in *DJ-1−/−* mice. Although we observed an intriguing increase in striatal TH fiber density and striatal DA in *DJ-1−/−* mice treated with LPS for 3 months, we did not observe significant changes in the cohorts receiving LPS for 6 months or for 3 months with a 3-month wait period. Some caution should therefore be exercised in interpreting the unexpected increases in striatal TH fiber density and striatal DA in the 3-month treatment group.

Although *DJ-1−/−* mice were reported to display normal numbers of nigral DA neurons in the SNpc, they were shown to have deficits in dopaminergic function [17] that are further accentuated when exposed to paraquat [26] and MPTP [25], suggesting a protective role for DJ-1 in mitochondrial function and/or against oxidative stress. In support of this molecular model, DJ-1 has been shown to translocate to mitochondria [20] in response to oxidative stress when key cysteine residues become oxidized [18], and to also interact with multifunctional regulators of transcription and RNA metabolism in the nucleus [42]. In contrast, we found that mice deficient in *DJ-1* displayed blunted oxidative stress levels in response to LPS treatment relative to wild-type mice (Figure 7), as measured by expression of Nrf2, HO-1 and iNOS. However, we found that expression of SOD-1 and SOD-2 was increased in saline-treated *DJ-1−/−* mice compared to saline-treated wild-type mice (Figure 7), which may reflect compensatory upregulation of certain anti-oxidants in the absence of DJ-1 and may have contributed to the attenuated expression of oxidative stress genes in response to LPS. Inflammatory stimuli have been reported to exacerbate the oxidative response of *DJ-1−/−* cells, and although we did not observe this in our study, we only reported responses after chronic LPS treatments (6 months) and did not have the acute response gene expression after 3 months of LPS injections to draw conclusions about the kinetics of the oxidative stress response. Additionally, gene expression was measured in lysates of whole brain sections and it is therefore likely that the differences we observed in oxidative gene expression in the *DJ-1−/−* mouse midbrain is attributed to other cell types in the brain, such as astrocytes or neurons which also express SOD-1 and SOD-2, for example. Interestingly, we also observed that saline-treated *DJ-1−/−* mice compared to saline-treated wild-type mice have increased CD45 in the midbrain. Although Iba-1 immunostaining indicated no robust changes in microglia number or activation status, this increased expression could be due to increased levels of other lymphocytes or neutrophils which also express CD45 [43]. Lastly, given that *DJ-1−/−* mice have been reported to have compensatory gene expression in mitochondrial enzymes [44], we considered and ruled out the possibility that compensatory increases in Parkin mRNA and protein expression accounted for the lack of vulnerability against inflammation-induced nigral degeneration.

In summary, we conclude that loss of DJ-1 function, unlike loss of Parkin function, does not confer increased vulnerability to the neurodegenerating effects of chronic inflammatory insults induced by LPS. Given the contrasting responses of *parkin−/−* and *DJ-1−/−* mice to repeated i.p. LPS injections, we conclude that Parkin and DJ-1 are likely to have distinct and non-overlapping roles in protecting the nigrostriatal pathway against inflammatory and neurotoxic insults that lead to degeneration.

## Abbreviations

ANOVA: analysis of variance; DA: dopamine; DMEM: Dulbecco’s modified Eagle’s medium; FBS: fetal bovine serum; HO-1: heme-oxygenase-1; HPLC: high-performance liquid chromatography; IFN: interferon; IL: interleukin; iNOS: inducible nitric oxide synthase; inTNF: intranasal administration of soluble tumor necrosis factor; i.p.: intraperitoneal; MPTP: 1-methyl-4-phenyl-1,2,3,6-tetrahydropyrindine; NE: nasal epithelia; NQO1: NAD(P)H quinone oxidoreductase-1; Nrf2: NF-E2 related factor; NSAIDS: nonsteroidal anti-inflammatory drugs; LPS: lipopolysaccharide; PBS: phosphate-buffered saline; PCR: polymerase chain reaction; PD: Parkinson’s disease; QPCR: quantitative polymerase chain reaction; rh-TNF: rhodamine-labeled human tumor necrosis factor; siRNA: small interfering RNA; SNpc: substantia nigra pars compacta; SOD: superoxide dismutase; TBS: Tris-buffered saline; TH: tyrosine hydroxylase; TNF: tumor necrosis factor; VTA: ventral tegmental area.

## Competing interests

The authors declare that they have no competing interests.

## Authors’ contributions

TAN: experimental design, food intake, dosing, behavior, tissue harvest, tissue processing, QPCR, HPLC, IHC, stereology, statistical analysis, paper editing. TF-C: experimental design, dosing, behavior, tissue harvest, tissue processing, IHC, stereology and striatal density, statistical analysis, paper editing. TNM: experimental design, dosing, behavior, tissue harvest, tissue processing, IHC, stereology, fluorescent microscopy, paper editing. KAR: animal colony, dosing, behavior, tissue harvest, perfusions, cryosectioning, stereology. MM: dosing, tissue processing for RNA, behavior, HPLC. BC: dosing, tissue harvest. JJH: IHC. IT: IHC, HPLC. MGT: experimental design, dosing, food monitoring, behavior, stereology, fluorescence microscopy, paper editing. MSG: experimental design, behavior, food monitoring, tissue harvests, stereology, striatal dissections, paper editing. All authors read and approved the final manuscript.

## Supplementary Material

Additional file 1: Figure S1 Schematic of intranasal TNF (inTNF) dosing paradigm in *DJ-1−/−* and wild type mice and effects on nigral DA neuron number and striatal DA. **A**, Schematic of experimental design. Wild type and *DJ-1−/−* mice were administered soluble murine TNF intranasally at the indicated concentrations or an equivalent volume of saline vehicle for the indicated times. Groups of mice were sacrificed as indicated for biochemical (striatal DA measurements by HPLC) and immunohistological (unbiased stereological estimate of nigral DA neuron number) analyses. **B**, Unbiased stereological analysis indicates that *DJ-1−/−* mice exposed to repeated doses of 0.5 ng TNF intranasally do not display a significant reduction of TH or NeuN immunopositive neurons in the SNpc. Error bars represent SEM, n=6-10 per group. Two-way ANOVA indicated no significant differences (TH: *p = 0.085*, NeuN: *p = 0.257*). **C,** Striatal dopamine (DA) was measured by HPLC with electrochemical detection and striatal DA turnover was calculated as the ratio of DA metabolites (DOPAC, HVA and 3-MT) to DA. Error bars represent SEM, n = 6–10 per group. Two-way ANOVA, Bonferroni’s post hoc **p < 0.01 compared to wild type saline (DA: F_(1,29)_= 22.07, *p* < 0.0001, DA turnover: not significant, *p* = 0.055). Click here for file

Additional file 2: Figure S2 Striatal levels of DA and metabolites for wild type and *DJ-1−/−* mice in the 3-month treatment group was measured by HPLC electrochemical detection. (DOPAC, HVA, and 3-MT were not significant by two-way ANOVA, *p >* 0.05 for all). Click here for file

Additional file 3: Figure S3 Neuroinflammatory and oxidative stress responses of isolated peritoneal macrophages from *DJ-1−/−* mice are similar to wild type. Primary peritoneal macrophages were isolated from wild type and *DJ-1−/−* mice and treated with 1 ug/mL LPS for 4 hrs. QPCR was performed for gene expression of TNF, iNOS, IL1b, NRF2, and NQO1. A two-way ANOVA was performed with Bonferroni’s post hoc at * for *p* < 0.05, ** for *p* < 0.01, and *** for *p* < 0.001 significance compared to the saline treatment. No genotype differences were detected (TNF: *p* = 0.95, iNOS: *p* = 0.33, IL1β: *p* = 0.60, NRF2: *p* = 0.54, NQO1: *p* = 0.09). Click here for file

## References

[B1] McGeer PL Schwab C Parent A Doudet D Presence of reactive microglia in monkey substantia nigra years after 1-methyl-4-phenyl-1,2,3,6-tetrahydropyridine administration Ann Neurol 2003 54 599 604 10.1002/ana.10728 14595649

[B2] Mrak RE Griffin WS Glia and their cytokines in progression of neurodegeneration Neurobiol Aging 2005 26 349 354 10.1016/j.neurobiolaging.2004.05.010 15639313

[B3] Wersinger C Sidhu A Inflammation and Parkinson’s disease Curr Drug Targets Inflamm Allergy 2002 1 221 242 10.2174/1568010023344580 14561187

[B4] Zhang L Dawson VL Dawson TM Role of nitric oxide in Parkinson’s disease Pharmacol Ther 2006 109 33 41 10.1016/j.pharmthera.2005.05.007 16005074

[B5] Tansey MG McCoy MK Frank-Cannon TC Neuroinflammatory mechanisms in Parkinson’s disease: potential environmental triggers, pathways, and targets for early therapeutic intervention Exp Neurol 2007 208 1 25 10.1016/j.expneurol.2007.07.004 17720159 PMC3707134

[B6] McGeer PL Itagaki S Boyes BE McGeer EG Reactive microglia are positive for HLA-DR in the substantia nigra of Parkinson’s and Alzheimer’s disease brains Neurology 1988 38 1285 1291 10.1212/WNL.38.8.1285 3399080

[B7] Cassarino DS Halvorsen EM Swerdlow RH Abramova NN Parker WD Jr Sturgill TW Bennett JP Jr Interaction among mitochondria, mitogen-activated protein kinases, and nuclear factor-kappaB in cellular models of Parkinson’s disease J Neurochem 2000 74 1384 1392 10737593 10.1046/j.1471-4159.2000.0741384.x

[B8] Banati RB Daniel SE Blunt SB Glial pathology but absence of apoptotic nigral neurons in long-standing Parkinson’s disease Mov Disord 1998 13 221 227 10.1002/mds.870130205 9539333

[B9] Hunot S Dugas N Faucheux B Hartmann A Tardieu M Debré P Agid Y Dugas B Hirsch EC FcepsilonRII/CD23 is expressed in Parkinson’s disease and induces, in vitro, production of nitric oxide and tumor necrosis factor-alpha in glial cells J Neurosci 1999 19 3440 3447 10212304 10.1523/JNEUROSCI.19-09-03440.1999 PMC6782235

[B10] Pavese N Gerhard A Tai YF Ho AK Turkheimer F Barker RA Brooks DJ Piccini P Microglial activation correlates with severity in Huntington disease: a clinical and PET study Neurology 2006 66 1638 1643 10.1212/01.wnl.0000222734.56412.17 16769933

[B11] Whitton PS Inflammation as a causative factor in the aetiology of Parkinson’s disease Br J Pharmacol 2007 150 963 976 17339843 10.1038/sj.bjp.0707167 PMC2013918

[B12] McCoy McCoy MK Martinez TN Ruhn KA Szymkowski DE Smith CG Botterman BR Tansey KE Tansey MG Blocking soluble tumor necrosis factor signaling with dominant-negative tumor necrosis factor inhibitor attenuates loss of dopaminergic neurons in models of Parkinson’s disease J Neurosci 2006 26 9365 9375 10.1523/JNEUROSCI.1504-06.2006 16971520 PMC3707118

[B13] McCoy MK Ruhn KA Martinez TN McAlpine FE Blesch A Tansey MG Intranigral lentiviral delivery of dominant-negative TNF attenuates neurodegeneration and behavioral deficits in hemiparkinsonian rats Mol Ther 2008 16 1572 1579 10.1038/mt.2008.146 18628756 PMC2670754

[B14] Sánchez-Pernaute R Ferree A Cooper O Yu M Brownell AL Isacson O Selective COX-2 inhibition prevents progressive dopamine neuron degeneration in a rat model of Parkinson’s disease J Neuroinflammation 2004 1 6 10.1186/1742-2094-1-6 15285796 PMC483059

[B15] Farrer MJ Genetics of Parkinson disease: paradigm shifts and future prospects Nat Rev Genet 2006 7 306 318 16543934 10.1038/nrg1831

[B16] Li HM Niki T Taira T Iguchi-Ariga SM Ariga H Association of DJ-1 with chaperones and enhanced association and colocalization with mitochondrial Hsp70 by oxidative stress Free Radic Res 2005 39 1091 1099 10.1080/10715760500260348 16298734

[B17] Goldberg MS Pisani A Haburcak M Vortherms TA Kitada T Costa C Tong Y Martella G Tscherter A Martins A Bernardi G Roth BL Pothos EN Calabresi P Shen J Nigrostriatal dopaminergic deficits and hypokinesia caused by inactivation of the familial Parkinsonism-linked gene DJ-1 Neuron 2005 45 489 496 10.1016/j.neuron.2005.01.041 15721235

[B18] Canet-Avilés RM Wilson MA Miller DW Ahmad R McLendon C Bandyopadhyay S Baptista MJ Ringe D Petsko GA Cookson MR The Parkinson’s disease protein DJ-1 is neuroprotective due to cysteine-sulfinic acid-driven mitochondrial localization Proc Natl Acad Sci U S A 2004 101 9103 9108 10.1073/pnas.0402959101 15181200 PMC428480

[B19] Zhang J Goodlett DR Peskind ER Quinn JF Zhou Y Wang Q Pan C Yi E Eng J Aebersold RH Montine TJ Quantitative proteomic analysis of age-related changes in human cerebrospinal fluid Neurobiol Aging 2005 26 207 227 10.1016/j.neurobiolaging.2004.03.012 15582749

[B20] Zhang L Shimoji M Thomas B Moore DJ Yu SW Marupudi NI Torp R Torgner IA Ottersen OP Dawson TM Dawson VL Mitochondrial localization of the Parkinson’s disease related protein DJ-1: implications for pathogenesis Hum Mol Genet 2005 14 2063 2073 10.1093/hmg/ddi211 15944198

[B21] Bonifati V Oostra BA Heutink P Unraveling the pathogenesis of Parkinson’s disease–the contribution of monogenic forms Cell Mol Life Sci 2004 61 1729 1750 15241550 10.1007/s00018-004-4104-1 PMC11138675

[B22] Bonifati V Rizzu P van Baren MJ Schaap O Breedveld GJ Krieger E Dekker MC Squitieri F Ibanez P Joosse M van Dongen JW Vanacore N van Swieten JC Brice A Meco G van Duijn CM Oostra BA Heutink P Mutations in the DJ-1 gene associated with autosomal recessive early-onset parkinsonism Science 2003 299 256 259 10.1126/science.1077209 12446870

[B23] Shen J Cookson MR Mitochondria and dopamine: new insights into recessive parkinsonism Neuron 2004 43 301 304 10.1016/j.neuron.2004.07.012 15294138

[B24] van der Brug MP Blackinton J Chandran J Hao LY Lal A Mazan-Mamczarz K Martindale J Xie C Ahmad R Thomas KJ Beilina A Gibbs JR Ding J Myers AJ Zhan M Cai H Bonini NM Gorospe M Cookson MR RNA binding activity of the recessive parkinsonism protein DJ-1 supports involvement in multiple cellular pathways Proc Natl Acad Sci U S A 2008 105 10244 10249 10.1073/pnas.0708518105 18626009 PMC2481328

[B25] Kim RH Smith PD Aleyasin H Hayley S Mount MP Pownall S Wakeham A You-Ten AJ Kalia SK Horne P Westaway D Lozano AM Anisman H Park DS Mak TW Hypersensitivity of DJ-1-deficient mice to 1-methyl-4-phenyl-1,2,3,6-tetrahydropyrindine (MPTP) and oxidative stress Proc Natl Acad Sci U S A 2005 102 5215 5220 10.1073/pnas.0501282102 15784737 PMC555037

[B26] Yang W Chen L Ding Y Zhuang X Kang UJ Paraquat induces dopaminergic dysfunction and proteasome impairment in DJ-1-deficient mice Hum Mol Genet 2007 16 2900 2910 10.1093/hmg/ddm249 17823202

[B27] Yamaguchi H Shen J Absence of dopaminergic neuronal degeneration and oxidative damage in aged DJ-1-deficient mice Mol Neurodegener 2007 2 10 10.1186/1750-1326-2-10 17535435 PMC1891294

[B28] Andres-Mateos E Perier C Zhang L Blanchard-Fillion B Greco TM Thomas B Ko HS Sasaki M Ischiropoulos H Przedborski S Dawson TM Dawson VL DJ-1 gene deletion reveals that DJ-1 is an atypical peroxiredoxin-like peroxidase Proc Natl Acad Sci U S A 2007 104 14807 14812 10.1073/pnas.0703219104 17766438 PMC1976193

[B29] Chen L Cagniard B Mathews T Jones S Koh HC Ding Y Carvey PM Ling Z Kang UJ Zhuang X Age-dependent motor deficits and dopaminergic dysfunction in DJ-1 null mice J Biol Chem 2005 280 21418 21426 10.1074/jbc.M413955200 15799973

[B30] Martinat C Shendelman S Jonason A Leete T Beal MF Yang L Floss T Abeliovich A Sensitivity to oxidative stress in DJ-1-deficient dopamine neurons: an ES- derived cell model of primary parkinsonism PLoS Biol 2004 2 e327 10.1371/journal.pbio.0020327 15502868 PMC521171

[B31] Mitsumoto A Nakagawa Y DJ-1 is an indicator for endogenous reactive oxygen species elicited by endotoxin Free Radic Res 2001 35 885 893 10.1080/10715760100301381 11811539

[B32] Waak J Weber SS Waldenmaier A Görner K Alunni-Fabbroni M Schell H Vogt-Weisenhorn D Pham TT Reumers V Baekelandt V Wurst W Kahle PJ Regulation of astrocyte inflammatory responses by the Parkinson’s disease-associated gene DJ-1 FASEB J 2009 23 2478 2489 10.1096/fj.08-125153 19276172

[B33] Frank-Cannon TC Tran T Ruhn KA Martinez TN Hong J Marvin M Hartley M Treviño I O’Brien DE Casey B Goldberg MS Tansey MG Parkin deficiency increases vulnerability to inflammation-related nigral degeneration J Neurosci 2008 28 10825 10834 10.1523/JNEUROSCI.3001-08.2008 18945890 PMC2603252

[B34] Venkateswaran A Repa JJ Lobaccaro JM Bronson A Mangelsdorf DJ Edwards PA Human white/murine ABC8 mRNA levels are highly induced in lipid-loaded macrophages. A transcriptional role for specific oxysterols J Biol Chem 2000 275 14700 14707 10.1074/jbc.275.19.14700 10799558

[B35] Kurrasch DM Huang J Wilkie TM Repa JJ Quantitative real-time PCR measurement of regulators of G-protein signaling (RGS) mRNA levels in mouse tissues Methods Enzymol 2004 389 3 15 15313556 10.1016/S0076-6879(04)89001-3

[B36] Frank TC Nunley MC Sons HD Ramon R Abbott LC Fluoro-jade identification of cerebellar granule cell and purkinje cell death in the alpha1A calcium ion channel mutant mouse, leaner Neuroscience 2003 118 667 680 10.1016/S0306-4522(03)00019-8 12710975

[B37] Abbott LC Jacobowitz DM Development of calretinin-immunoreactive unipolar brush-like cells and an afferent pathway to the embryonic and early postnatal mouse cerebellum Anat Embryol (Berl) 1995 191 541 559 7677260 10.1007/BF00186743

[B38] Paxinos G Franklin KBJ The Mouse Brain in Stereotaxic Coordinates 2001 2 San Diego, CA: Academic

[B39] Braak H Del Tredici K Rüb U de Vos RA Jansen Steur EN Braak E Staging of brain pathology related to sporadic Parkinson’s disease Neurobiol Aging 2003 24 197 211 10.1016/S0197-4580(02)00065-9 12498954

[B40] Larsen NJ Ambrosi G Mullett SJ Berman SB Hinkle DA DJ-1 knock-down impairs astrocyte mitochondrial function Neuroscience 2011 196 251 264 21907265 10.1016/j.neuroscience.2011.08.016 PMC3490195

[B41] Kahle PJ Waak J Gasser T DJ-1 and prevention of oxidative stress in Parkinson’s disease and other age-related disorders Free Radic Biol Med 2009 47 1354 1361 10.1016/j.freeradbiomed.2009.08.003 19686841

[B42] Xu J Zhong N Wang H Elias JE Kim CY Woldman I Pifl C Gygi SP Geula C Yankner BA The Parkinson’s disease-associated DJ-1 protein is a transcriptional co-activator that protects against neuronal apoptosis Hum Mol Genet 2005 14 1231 1241 10.1093/hmg/ddi134 15790595

[B43] Hermiston ML Zikherman J Zhu JW CD45, CD148, and Lyp/Pep: critical phosphatases regulating Src family kinase signaling networks in immune cells Immunol Rev 2009 228 288 311 10.1111/j.1600-065X.2008.00752.x 19290935 PMC2739744

[B44] Pham TT Giesert F Röthig A Floss T Kallnik M Weindl K Hölter SM Ahting U Prokisch H Becker L Klopstock T Hrabé de Angelis M Beyer K Görner K Kahle PJ Vogt Weisenhorn DM Wurst W DJ-1-deficient mice show less TH-positive neurons in the ventral tegmental area and exhibit non-motoric behavioural impairments Genes Brain Behav 2010 9 305 317 10.1111/j.1601-183X.2009.00559.x 20039949

